# A patient with Sagliker syndrome who underwent parathyroidectomy: A case report and literature review 

**DOI:** 10.5414/CNCS111888

**Published:** 2026-05-11

**Authors:** Jianping Ren, Xinyu Li, Baoyu Zhao, Deguang Wang, Xuerong Wang

**Affiliations:** Department of Nephrology, The Second Affiliated Hospital of Anhui Medical University, Hefei, Anhui, China; * These authors contributed equally to this work.

**Keywords:** Sagliker syndrome, chronic kidney disease, secondary hyperparathyroidism, parathyroidectomy

## Abstract

Background: Sagliker syndrome (SS) is a rare yet severe complication of end-stage renal disease that is predominantly observed in patients who have undergone prolonged hemodialysis and develop secondary hyperparathyroidism (SHPT). SS is characterized by progressive craniofacial and maxillofacial bone deformities, such as mandibular widening, increased interdental spacing, and neuropsychiatric symptoms including anxiety, depression, and cognitive dysfunction. These manifestations significantly impair the quality of life of affected individuals. Case report: We report the case of a 42-year-old woman undergoing regular hemodialysis who was diagnosed with SHPT and SS. The patient was admitted to the hospital with a 3-year history of skeletal deformity. On December 29, 2012, she underwent total parathyroidectomy (PTx) with some parathyroid tissue transplanted to the left forearm. Over 12 years of regular follow-up, we found that clinical symptoms, such as facial deformity and bone pain, and laboratory indicators, including corrected serum calcium and phosphorus levels, significantly improved. Amazingly, the skull, which was altered in a “salt and pepper” pattern, improved significantly. Conclusion: After PTx in patients with SS, clinical symptoms and related laboratory indicators can be significantly improved, which is conducive to enhancing patient prognosis. Therefore, PTx should be performed in such patients as early as possible. However, further research is needed to develop a more standardized treatment plan for SS.

## Introduction 

Secondary hyperparathyroidism (SHPT) refers to excessive secretion of parathyroid hormone (PTH) or hyperplasia of the parathyroid tissue due to mineral metabolism disorder in patients with chronic kidney disease (CKD). It constitutes a critical component and common complication of chronic kidney disease-mineral and bone disorder (CKD-MBD). In rare cases with severe progression, it may evolve into Sagliker syndrome (SS). SS is commonly found in patients with end-stage renal disease, especially in those who have been treated with long-term hemodialysis. In 2004, SS was first reported and named by a team led by Professor Sagliker, a nephrologist from Turkey. The syndrome is characterized by severe craniomaxillofacial skeletal deformities, metabolic disorders, and neuropsychiatric symptoms. Herein, we report the case of a patient with SS who underwent parathyroidectomy (PTx). After 12 years of regular follow-up, we observed that the patient exhibited significant relief from facial deformity and bone pain symptoms after surgical removal of the parathyroid gland and autologous left abdominal wall transplantation, leading to an improvement in quality of life (QoL). Additionally, the “salt and pepper” changes in skull abnormalities were markedly ameliorated. Calcium, phosphorus, and PTH levels remained within the normal range. A review of the literature, including general characteristics, risk factors, and treatment options of SS, is summarized to provide reference for early clinical diagnosis and treatment to improve patient QoL. 

## Case report 

A 42-year-old Chinese woman visited our hospital on December 18, 2012, with a history of elevated serum creatinine levels (for 17 years) and maxillofacial deformities (for 3 years). When the patient came to our hospital 17 years prior, she had been diagnosed with CKD at a local hospital due to elevated serum creatinine and urea nitrogen levels during a physical examination and received conservative drug treatment. Five years later, the patient gradually developed symptoms, such as nausea and vomiting. Serum creatinine levels increased to 1,158 μmol/L. She was diagnosed with CKD stage 5 and underwent peritoneal dialysis. After 3 years of unsuccessful treatment, the patient switched to hemodialysis therapy. During 3 years of hemodialysis, the patient experienced maxillofacial deformity and sternal lordosis, as well as bilateral knee, ankle, and heel pain. The symptoms gradually worsened, and the patient’s height decreased by 33 cm (162 cm to 129 cm). 

The patient had no past or family history of obvious abnormalities. Physical examination upon admission showed obvious maxillofacial deformity; difficulty in lip closure; widening of the gap between the teeth; short neck; distended jugular veins; swollen chest, spine, and bilateral distal phalanges; and limited movement of the hip and knee joints. Additionally, a continuous tremor was felt in the arteriovenous fistula in the right forearm and a 3/6 systolic murmur was heard upon auscultation at that specific location. 

Laboratory tests showed that creatinine, urea nitrogen, and cystatin-C levels were increased, indicating the patient’s renal function was impaired; corrected serum calcium and phosphorus levels were elevated at 3.22 mmol/L and 1.85 mmol/L, respectively; total PTH levels were significantly elevated (> 2,500 pg/mL); and alkaline phosphatase (ALP) levels were also increased (up to 2,340 U/L). Results suggested the patient had CKD-MBD. Although the patient was not anemic, an albumin level of 28.4 g/L suggested poor nutrition. 

Parathyroid ultrasound showed a hypoechoic mass behind the lower pole of the bilateral thyroid glands. A computed tomography scan of the cervical chest indicated a lump in the dorsal segment of the right lobe of the thyroid, with a maximum cross-section of ~ 20 × 15 mm, and thoracic vertebral deformity, with osteoporosis in the maxillofacial region and sternum. Dual-phase methoxyisobutylisonitrile parathyroid imaging showed radioactive retention at the lower pole of the bilateral thyroid glands, indicating HPT. Multiple bone abnormalities in the maxillofacial region and chest suggested metabolic bone disease. Plain skull radiographs showed abnormal changes in the skull and maxillofacial bones, bulging of the maxillofacial bones, increased density, multiple patchy high-density and cystic low-density mixed changes, and bone destruction. Cardiac color Doppler ultrasound showed calcification of the aortic and mitral valves. 

Based on the combination of clinical manifestations and physical examination, the patient was diagnosed with end-stage renal disease on maintenance hemodialysis and SHPT with SS. In terms of treatment, the patient was administered maintenance hemodialysis 3 times a week, erythropoietin to correct renal anemia, calcitriol to treat HPT, and other symptomatic treatments after admission. Due to refractory SHPT and severe bone deformity, the patient underwent total PTx with autotransplantation (tPTx+AT) under general anesthesia on December 29, 2012. Postoperative parathyroid pathology revealed massive hyperplasia of parathyroid tissue. After surgery, serum calcium levels decreased to 1.32 mmol/L, and oral and intravenous calcium supplementation, oral calcitriol, was given to promote calcium absorption. Ten days after the operation, serum calcium levels were stabilized (> 1.80 mmol/L) with oral calcium supplementation. During the hospitalization period, the patient’s postoperative intact PTH (iPTH) levels remained within the normal range (18.8 – 42.3 pg/mL). Serum phosphorus levels also returned to normal (1.44 mmol/L). The patient had no bleeding, coughing when drinking water, or hoarseness and reported the pain was significantly relieved in both knee and ankle joints and heels. The aforementioned results indicate the operation was successful. 

**Figure 1:**
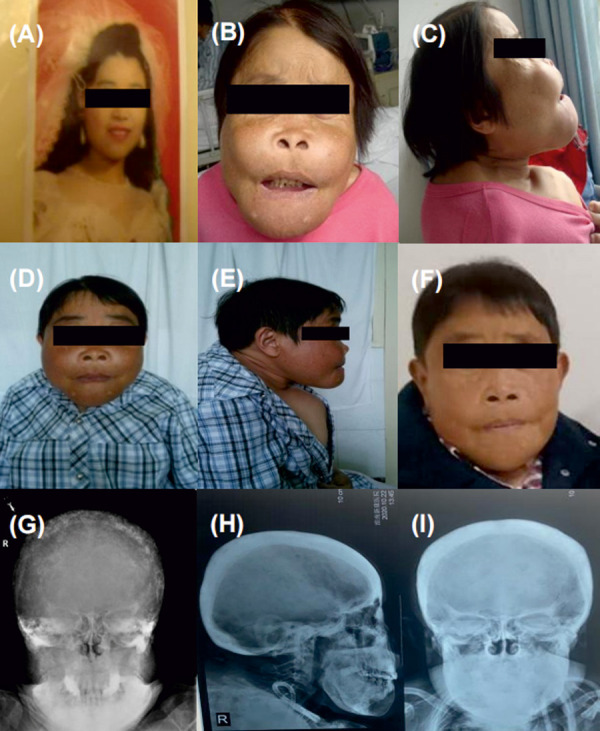
Comparison of facial deformity and skull “salt and pepper” appearance in patient with Sagliker syndrome before and after surgery. A: The patient’s normal facial appearance at the age of 20. B, C: Lion-like facial appearance before parathyroidectomy, characterized by craniofacial deformities, inability to fully close the lips, dental abnormalities, severe thoracic and spinal deformities, and a reduction in height from 162 cm to 129 cm. D, E: One year after parathyroidectomy, craniofacial deformity improved and height increased to 133 cm. F: Eight years after parathyroidectomy, craniofacial deformity improved even more significantly. G: Pre-parathyroidectomy frontal skull X-ray showed cranial thickening, accompanied by sclerosis and lytic changes, increased bone density, and bone destruction with “salt and pepper” appearance. H, I: Significant relief in skull X-rays observed at 8 years post-parathyroidectomy.

At the most recent follow-up (~ 12 years post operation), the patient continues to require regular hemodialysis. Her current medication regimen during dialysis and in the outpatient setting includes: amlodipine, 5 mg/day, and carvedilol, 10 mg/day, for hypertension; sevelamer, 0.8 g 3 times daily, for hyperphosphatemia; and erythropoietin, 5,000 units 3 times per week, for renal anemia. During the years of follow-up, the patient’s facial deformity, skull plain bone destruction, and many laboratory indicators significantly improved after surgery (Figures 1, 2A). Corrected serum calcium and phosphorus levels were maintained within the normal range (Figure 2B). Blood pressure was maintained above 120/90 mmHg, and hemoglobin levels were within the normal range (< 5,000 IU of erythropoietin per week). After the operation, the patient’s bone pain was rapidly relieved, and the bone pain score decreased from 5 (pre-operation) to 3 (post-operation) (Figure 3). Approximately 12 years after the surgery, the patient’s bone pain completely disappeared. Her weight and height increased from 33 kg (pre-operation) to 41 kg (present) and 129 cm (pre-operation) to 133 cm (present), respectively. The Kidney Disease Quality of Life-36 (KDQOL-36) scale was used to quantitatively assess patient QoL, and the results were compared and analyzed with those of the preoperative questionnaire. Results showed the scores of all dimensions of the KDQOL-36 scale were significantly higher than those before the operation, indicating that surgery significantly improved the patient’s QoL in multiple dimensions (Table 1). 

We recommended the patient undergo follow-up examinations, such as echocardiography, upright abdominal radiography, and bone density testing, based on her physical condition. Results showed aortic and mitral valve calcifications had completely disappeared, and no obvious calcification was observed in the abdominal aorta. Bone density test indicated reduced bone mass, which had not yet progressed to osteoporosis. These results suggest PTx can improve the long-term prognosis for patients with SS. During the 6-month period preceding the most recent follow-up, the patient developed intermittent hyperphosphatemia. The management approach included intensified dietary education with a strict limitation of daily phosphorus intake (< 800 – 1000 mg/day) and prescription of sevelamer 0,8 g three times daily, a non-calcium-based phosphate binder. Concurrently, to prevent persistent low-turnover bone disease following surgery and support skeletal health, paricalcitol, an active vitamin D analog, was administered. Treatment was initiated at a dose of 2 μg/day and subsequently adjusted to 1- to 3-month intervals based on serum iPTH, calcium, and phosphorus levels, with the objective of maintaining iPTH levels within 1.5 to 3 times the upper limit of normal while avoiding both hypercalcemia and hyperphosphatemia. Unfortunately, due to the patient’s refusal to undergo further re-examinations, the identification and screening of potential complications poses a significant challenge. 

## Literature review 

By reviewing the relevant literature over the past 20 years, we summarized the clinical data of 24 patients with SS (Table 2) [1, 2, 3, 4, 5, 6, 7, 8, 9]. In cases with known primary diseases, 11 patients (46%) had chronic glomerulonephritis and 2 patients (8%) had polycystic kidney disease. A total of 21 patients (87%) chose surgical treatment, of which 17 underwent tPTx. After surgery, almost all patients showed improvements in biochemical indicators and symptoms, such as bone pain. 

## Discussion 

As early as 1953, Cohen and Diamond [1] reported a case of facial deformity caused by severe SHPT in a CKD patient. However, this did not attract significant attention. In 2004, Sagliker et al. [10] collected 25 cases of maintenance hemodialysis patients with SHPT in Turkey. All patients had special facial deformities, mainly characterized by changes in the palate and mandible, skull abnormalities, and shortened stature. Therefore, Sagliker officially named this type of clinical manifestation SS [2]. Severe skeletal deformities can cause pain and limit mobility in patients. Skull deformities can cause “lion-like” facial changes in patients. These symptoms significantly reduce patient QoL and adversely affect their physiology and psychology. Patients are likely to develop a series of psychological problems due to disfiguring changes in their appearance, including depression. The patient reported in our case exhibited characteristic cranial bone alterations and severe bone pain symptoms during progressive development of the disease. Upon admission, she had a 12-item Short Form Survey Mental Composite Score of 37.16 as assessed by the KDQOL-36 scale, which was significantly lower than the normal range, indicating a high risk of severe mental health problems for the patient. 

However, the precise pathogenesis of SS remains incompletely elucidated. Current evidence suggests that its development is closely associated with profound disturbances in mineral and bone metabolism secondary to SHPT. Although the etiology of SS has yet to be fully defined, Demirhan et al. [11] conducted a 6-year global investigation involving 40 patients diagnosed with SS, performing genetic analyses on blood samples obtained from both the patients and their first-degree relatives. Their findings indicated the molecular genetic underpinnings of SS are primarily attributable to various somatic mutations in genes, such as *GNAS1*, *FGF23*, and *FGFR3*. In particular, loss-of-function or functionally abnormal mutations in *GNAS1* may impair G protein-coupled signaling pathways, thereby contributing to aberrant skeletal responses to SHPT. Mutations in *FGF23* can compromise its essential role in phosphate homeostasis under the background of CKD, further aggravating hyperphosphatemia and the severity of SHPT. Meanwhile, alterations in *FGFR3* directly disrupt key regulatory pathways involved in bone growth and development, providing a genetic basis for the characteristic skeletal deformities observed in SS, including craniofacial remodeling and limb abnormalities. Under the “second hit” imposed by CKD-MBD as CKD progresses, these genetic aberrations may act synergistically and become amplified, ultimately leading to the distinctive and severe skeletal deformities and multisystem involvement that define SS. While elucidation of these molecular mechanisms holds considerable scientific value, comprehensive genetic screening was not performed in the present case due to constraints related to accessibility, financial cost, and its limited relevance to the current therapeutic strategy. Furthermore, the patient, who currently resides in another location, has temporarily declined additional genetic evaluation. Future investigations utilizing next-generation sequencing in larger cohorts of patients with SS may provide further insight into its underlying genetic architecture. 

In addition, many existing studies have shown that long dialysis vintage, high ALP and iPTH levels, low albumin levels, and heart valve and abdominal aortic calcifications are factors that affect and may be risk factors for SS [12, 13]. However, the exact cause and pathogenesis of SS requires further research. The patient in our study had been undergoing regular dialysis for 12 years when admitted to the hospital. ALP and iPTH levels were significantly elevated, while albumin levels were decreased. In addition, the patient presented with cardiac valve calcification. These factors are considered to have contributed to the occurrence of SS. 

Currently, there is no unified standard diagnosis for SS. Based on SHPT in maintenance hemodialysis patients, the diagnosis relies on clinical symptoms, which include: severe maxillofacial changes that manifest as distorted facial appearance, maxillary protrusion, skull deformity, and nasal bone destruction; oral lesions, which may cause upper and lower jaws to be unable to bite, widened teeth, and benign soft tissue tumor-like hyperplasia; significant shortening in height; bone deformities in other parts of the body, and even spontaneous pathological fractures, including X- or O-shaped legs in the lower limbs and scapular deformity; and hypertrophy of the distal phalanges of fingers [14]. 

Based on previous reports and cases from our center, characteristics of SS can be summarized as follows. Many patients received regular long-term dialysis, but key biochemical indicators, such as iPTH, calcium, phosphorus, and ALP, were not continuously monitored during dialysis. As a result, SHPT often progressed to advanced and severe SHPT owing to a lack of timely intervention [15]. Notably, the primary disease in most patients was chronic glomerulonephritis. Additionally, SS patients often presented with special changes, mainly involving facial bone deformities, including abnormal morphology of the palatine bone and mandible, and some had benign oral soft tissue tumors [2]. Skull X-ray examination showed significantly thickened and denser bone plates with “salt and pepper” appearance; in severe cases, bones were expanded and had uneven density, leading to obvious cranial deformities and lion-like facial changes. Specific cranial manifestations included increased volume and density of the maxilla and mandible, widened interdental spaces, absorption of the alveolar bone plate, and blurred edges [16, 17]. At the same time, patients usually had multiple skeletal deformities throughout the body, such as shortened height, facial protrusion, and pseudo-clubbing of the fingers [14]. Moreover, patients often had varying degrees of neuropsychiatric symptoms, including headache, insomnia, anxiety, depression, and paresthesia [18, 19, 20], and could be accompanied by hearing loss [21]. The aforementioned characteristics are consistent with the case we reported with the exception of any abnormal manifestations in terms of hearing. 

The core strategy for treating SS is the same as the medical and surgical management of SHPT, including lowering serum phosphorus (a low phosphorus diet, use of phosphate binders, and dialysis), supplementing serum calcium (use of vitamin D and calcium supplements), and PTx [22, 23]. PTx is the first choice in treating SS. Surgery can provide timely control and improve the patient’s symptoms. Scholars have concluded that PTx can improve clinical symptoms and long-term prognosis of patients with SS, but bone deformities cannot be completely restored [3]. Currently, the main surgical methods are subtotal PTx, tPTx, and tPTx+AT. Subtotal PTx removes 3.5 glands with a low degree of hypoparathyroidism and low incidence of hypocalcemia after surgery; however, the residual parathyroid glands may undergo proliferation once more, leading to the recurrence of SHPT. TPTx removes all parathyroid glands, does not require autologous transplantation, and has a low recurrence rate. The literature reports that tPTX+AT is more effective in controlling long-term HPT, but there is still a risk for long-term hypocalcemia [24]. Patients with HPT almost certainly develop hypocalcemia after surgery, which can be corrected with intravenous or oral calcium supplementation [4]. Furthermore, previous reports have indicated that successful kidney transplantation can treat severe SHPT at its source by restoring renal function and correcting the uremic environment, thereby blocking the fundamental pathological and physiological processes that drive the progression of SS. It can effectively cause a significant decline in PTH levels and is expected to prevent the further development of skeletal deformities [10, 25]. However, if the transplantation fails (due to acute rejection or poor patient compliance) and after the patient returns to dialysis, rebound HPT can occur, which may instead accelerate the progression of SS [1]. In summary, kidney transplantation is a highly promising fundamental treatment option for SS patients, but its efficacy depends largely on the long-term functional stability of the transplanted kidney. Future management strategies should focus on individualized assessment and actively explore sequential treatment regimens combining PTx with kidney transplantation to maximize the long-term prognosis and patient QoL. Due to various factors, including scarcity of donors, kidney transplantation was not performed during the 12-year follow-up period in the patient presented in our case. 

In 2000, Sagliker et al. [26] initiated a multicenter study involving 40 patients (26 men, 14 women) with CKD-associated SS from Turkey, India, Romania, Egypt, Malaysia, Tunisia, and China. This international study analyzed the clinical characteristics, and the findings revealed that due to a prolonged lack of standardized treatment, these patients exhibited irreversible facial deformities (e.g., lion-like appearance), skeletal abnormalities (e.g., cranial nervous system anomalies, pathological fractures), and severe psychological issues. This study emphasized the critical importance of early intervention in specialized medical centers to prevent disease progression and improve prognosis. The patient reported in our case has not yet developed significant neuropsychiatric symptoms, which is considered to be related to her long-term standardized dialysis and timely surgical treatments. In the 12 years following surgery, all relevant indicators remained within the normal range without any significant abnormalities. Bone pain symptoms were completely relieved and no complications, such as skin itching or restless legs syndrome, were observed. The lion-like changes in the craniofacial region not only ceased to progress but also gradually showed an improving trend, indicating the possibility of restoring a normal appearance. Results from the KDQOL-36 scale clearly revealed the pattern and extent of the benefits that surgery brought to the patient. In 2020, a case report described a patient who had been on maintenance hemodialysis for 7 years and, 11 years after onset of SHPT, developed the characteristic craniofacial deformities of SS [25]. Subsequently, the patient underwent PTx+AT. Delayed PTx resulted in permanent functional and cosmetic changes in the patient’s face and hands as well as prolonged hospitalization. Therefore, clinicians should prioritize the screening and management of high-risk patients with maintenance hemodialysis and poorly controlled SHPT, performing PTx before SS symptoms manifest. Although the incidence of SS accounts for only 0.5% of maintenance hemodialysis patients [10], mortality and disability rates among these patients are significantly higher than those with other SHPT. In addition, because of poor patient compliance and high treatment costs, SS diagnosis and treatment remain a serious problem, which is a huge burden for patients, families, and even society. In summary, we found that early intervention of SHPT effectively halted disease progression. Particular emphasis should be placed on the dynamic monitoring of PTH levels and bone morphometric assessment in dialysis patients and conducting surgical treatment at the optimal time. This case report provides data for one of the longest follow-up periods for a patient with SS to date (12 years). This long-term longitudinal observation not only confirms the lasting efficacy of the surgery in maintaining biochemical controls but also documents the rare partial reversibility of craniofacial deformities, providing crucial insights into the natural history of the disease and the long-term prognosis of surgical intervention. A limitation of this study is that it is a single case report and lacks control data from patients with SS who did not undergo PTx. However, given the rarity of this disease, such prospective controlled studies are difficult to conduct. Furthermore, it is essential to investigate new drugs and establish standardized surgical treatment protocols. 

## Ethical approval 

This study was approved by the Ethics Committee of the Second Affiliated Hospital of the Anhui Medical University (PJ-YX2020-006). Written informed consent was obtained from the patient to participate in this study, which was conducted in accordance with the basic principles of the Declaration of Helsinki. 

## Informed consent 

This case report and attached patient images were approved by the informed consent of the patient. 

## Authors’ contributions 

Jianping Ren and Xinyu Li: study conception and design, manuscript drafting. Baoyu Zhao: data acquisition. Deguang Wang: clinical management and data interpretation.Xuerong Wang: study supervision, critical revision of the manuscript, and final approval. All authors contributed to the article and approved the submitted version. 

## Funding 

This work was funded by the Health Research Program of Anhui Province (AHWJ2023A20134), Research Fund of Anhui Institute of Translational Medicine (2023zhyx-B09), and Quality Project of Universities in Anhui Province (2024aijy580). 

## Conflict of interest 

The authors declare that the research was conducted in the absence of any commercial or financial relationships that could be construed as potential conflicts of interest. 

**Figure 2A.**
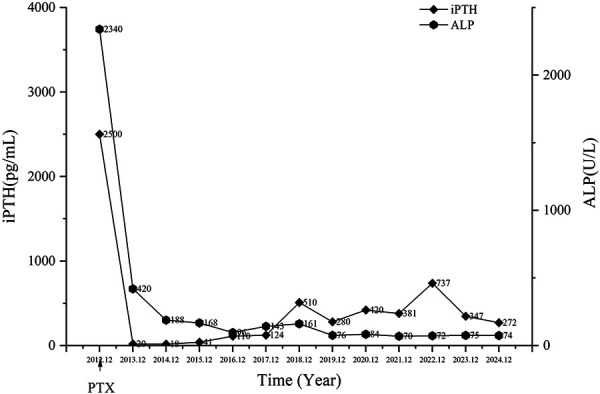
Patient’s serum intact parathyroid hormone (iPTH) and alkaline phosphatase (ALP) levels from 2012 to 2024.

**Figure 2B.**
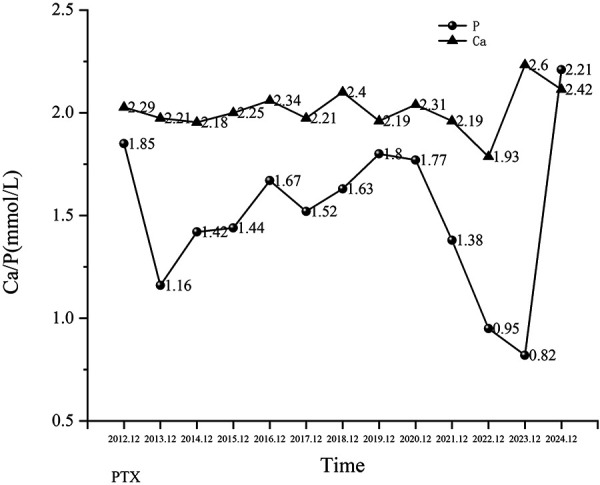
Patient’s corrected serum calcium (Ca) and serum phosphorus (P) levels from 2012 to 2024.

**Figure 3.**
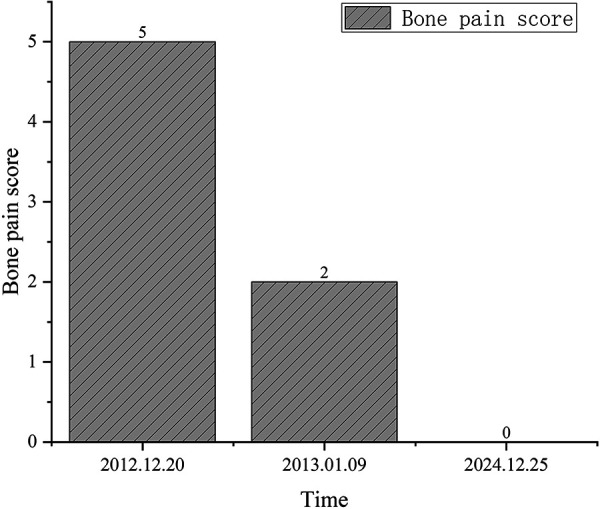
Preoperative and current bone pain scores of patient.

**Table 1.** Scores of each dimension of the Kidney Disease Quality of Life-36 scale of the patient before the operation and during follow-up.

**Dimension**	**Before operation**	**During follow-up**	**Absolute difference**
Symptom/problem list	41.67	89.58	+47.91
Effects of kidney disease	21.88	65.63	+63.24
Burden of kidney disease	6.25	31.25	+25.00
12-item Short Form Survey Physical Composite Score	22.30	37.39	+15.09
12-item Short Form Survey Mental Composite Score	37.16	58.14	+20.98

**Table 2.** Summary of all reported cases.

**Factor**	**Number**	**%**
Total cases	24	
Gender		
Female	15	63
Male	9	37
Age (years)		
< 30	5	21
30 – 49	7	29
50 – 69	2	8
Unknown	10	42
Primary diseases		
Chronic glomerulonephritis	11	46
Polycystic kidney disease	2	8
Unknown	11	46
Dialysis method		
Hemodialysis	18	75
Unknown	6	25
Treatment		
Total parathyroidectomy	17	71
Subtotal parathyroidectomy	2	8
Total parathyroidectomy with autotransplantation	2	8
No surgery	3	13
